# The role of Nature‐based Solutions in disaster resilience in coastal Jamaica: current and potential applications for ‘building back better’

**DOI:** 10.1111/disa.12539

**Published:** 2022-06-13

**Authors:** Simone Lee, Giselle Hall, Camilo Trench

**Affiliations:** ^1^ PhD Candidate, Centre for Environmental Management University of the West Indies Jamaica; ^2^ Independent Consultant Jamaica; ^3^ Chief Scientific Officer, Centre for Marine Sciences University of the West Indies

**Keywords:** coral, disaster resilience, Jamaica, Nature‐based Solutions

## Abstract

Jamaica, like most Small Island Developing States around the world, is at high risk of coastal hazards due to its exposure to tropical storms, high levels of coastal development, vulnerable coastal communities, and the predicted impacts of climate change. Environmental degradation has been linked to increased vulnerability to disasters. Nature‐based Solutions, although not formally present in the literature, are being implemented at various scales in Jamaica. This paper presents an overview of three marine and coastal Nature‐based Solutions being utilised in the country: protected area management (Special Fishery Conservation Areas); mangrove restoration; and coral restoration. The paper briefly reviews their current application in Jamaica before arguing that these conservation projects that traditionally focused on biodiversity have co‐benefits as Nature‐based Solutions for disaster resilience. The paper closes by outlining several research objectives that should be explored in the future to further the implementation of Nature‐based Solutions for disaster resilience in Jamaica.

## Introduction: Jamaica's vulnerability to coastal hazards

Jamaica, like most Small Island Developing States, is at high risk of coastal hazards due to its exposure to tropical storms, high levels of coastal development, vulnerable coastal communities, and the predicted impacts of climate change (Stephenson and Jones, 2017; Mycoo, 2018). In fact, coastal hazards, such as hurricanes and storms, account for approximately 75 per cent of hazards with natural causes in the Caribbean region (Collymore, 2011; Carby, 2018). Coastal areas host around 70 per cent of Jamaica's population, as well as some 56 per cent of its economic assets, such as airports, harbours, and tourism infrastructure. Consequently, coastal hazards are a very serious social and economic concern, posing significant challenges to disaster recovery and redevelopment (Richards, 2008). Eleven major storms affected Jamaica between 1988 and 2011, severely impacting people and property; storm surge reached as high as three to six metres in areas of the capital, Kingston (Robinson and Khan, 2011). Jamaica has experienced several major hurricanes, including Irma and Maria in September 2017, which caused more than USD 2 billion in losses (Planning Institute of Jamaica, 2004).

Sea‐level rise (SLR) is also a constant threat to coastal areas. The Intergovernmental Panel on Climate Change's 2013 report highlights the fact that, globally, there was a total SLR of ∼0.19 metres ± 0.02 metres between 1901 and 2010, that is, at a rate of 1.7+0.2 millimetres per annum (Stocker et al., 2013). Recent global studies, such as Yi et al. (2015), also underscore the increasing danger of accelerated rates of SLR due to sustained high global and regional temperatures. Caribbean SLR trends have historically followed global trends (Climate Studies Group, Mona, 2015). In Jamaica, total SLR has been calculated to be 1.66 millimetres per year, with a projected increase in total SLR of 0.87–0.9 metres by the end of the twenty‐first century. The impact of SLR and intensified storm surge is anticipated to be high for Jamaica, with 29 per cent of the coastal population exposed, and potential losses of coastal gross domestic product projected to exceed 27 per cent (Dasgupta et al., 2009). The effects in some areas, such as shoreline retreat in Negril, have exceeded predictions: a 16‐centimetre retreat as compared to the proposed seven centimetres (Robinson and Khan, 2011). Stephen and Jones (2017) note the negative impacts of SLR on Alligator Pond on the southern coast of Jamaica. The increased incidence of SLR and storm surge could lead to the displacement of at least 25 per cent of Jamaicans living in vulnerable areas, according to coastal flooding models (Climate Studies Group, Mona, 2015), and destroy critical infrastructure, such as airports and road networks. The predicted trends and consequences of climate change are expected to exacerbate Jamaica's vulnerability to environmental hazards.

Another factor contributing to disaster vulnerability is the condition of the associated habitats (Arkema et al., 2013; Ali et al., 2018). The Caribbean has lost between 24 and 28 per cent of mangrove cover since the 1980s, as a result of real estate competition for income generation and human infrastructure such as industry, tourism, mariculture, and civil construction (Torres Ortega et al., 2019). More specifically, an analysis of limited data suggests that in 2013, there were 9,800 hectares of mangrove forest in Jamaica; more than 770 hectares of mangrove forest was lost between 1996 and 2006 (Torres Ortega et al., 2019). In the last *Coral Reef Health Status Report for Jamaica* (National Environment and Planning Agency, 2021), the majority (41 per cent) of sites assessed were considered to be ‘poor’, whereas a substantial proportion (36 per cent) were considered to be critical; no assessed sites were considered to be ‘good’ according to the Coral Reef Health Index. This is not uncommon in the Caribbean region and hence can make the islands more vulnerable to other pressures (Mumby, Hastings, and Edwards, 2007). Coral bleaching events have also been known to impact negatively on Jamaica's and the wider Caribbean's coral reefs (Gates, 1990; Eakin et al., 2010). In addition, herbivorous and commercial fish populations exhibited an overall decline (78 and 97 per cent, respectively) between 2011 and 2020 (National Environment and Planning Agency, 2021).

Disasters themselves can also contribute to the declining condition of coastal ecosystems. Hurricanes in 1980 and 1988 demolished coral zones on the north coast of Jamaica, and any passage of a hurricane within a range of approximately 65 kilometres can cause significant damage (Björn et al., 1986; Woodley, 1991). Repeated hurricane and storm tracks have caused beach erosion in different areas, such as in Long Bay in Negril and along parts of the south coast of Jamaica (McKenzie, 2012; Campbell and Lee, 2021). Mangrove wetland forests are also affected by storms (Smith et al., 2009), with different stands in Jamaica suffering the impacts of hurricanes and other events (Wunderle, Lodge, and Waide, 1992; Henry, Webber, and Webber, 2018).

### Nature's role in ‘building back better’

Declines in the extent and condition of habitats and ecosystems affect their functionality and their ability to provide ecosystem services or benefits to people (Arkema et al., 2013; Ali et al., 2018). The concept of ecosystem services has been around since the 1970s, encompassing several definitions and categories, including those of: the Millennium Ecosystem Assessment; The Economics of Ecosystems and Biodiversity Project; the Common International Classification of Ecosystem Services; and the Intergovernmental Science‐Policy Platform on Biodiversity and Ecosystem Services' ‘Nature's Contributions to People’ (Reid et al., 2005; Sukhdev et al., 2010; Vihervaara, Rönkä, and Walls, 2010; Haines‐Young and Potschin, 2018; Kadykalo et al., 2019). Although there are differences between the classifications, broadly, they speak to the role of nature in providing provisioning, regulating, cultural, and supporting services to humans.

The concept of ecosystem services underpins the emerging term ‘Nature‐based Solutions’ (NbS), which is defined by the International Union for Conservation of Nature and Natural Resources (IUCN) as: ‘actions to protect, sustainably manage, and restore natural and modified ecosystems that address societal challenges effectively and adaptively, simultaneously providing human well‐being and biodiversity benefits’.^1^ These NbS can cover a wide array of activities, including ecosystem restoration, integrated coastal zone management, and area‐based conservation, while tackling myriad societal challenges. An increasingly important aspect of NbS is their ability not only to provide services to meet the challenges for which they were designed, but also to generate additional co‐benefits (Giordano et al., 2020). NbS have been employed to minimise climate‐related risks to people and property, playing an important part in disaster risk reduction (DRR), resilience, and management (Young et al., 2019; Giordano et al., 2020). They can also build ecological resilience through improved physical conditions and ecosystem health and services, social resilience through improved mental health, well‐being, social cohesion, and networks, and economic resilience through the avoidance of damage, indirectly improving access to financial resources saved from offsetting losses due to climate‐related disasters (Narayan et al., 2016; Enzi et al., 2017; Mabon, 2019). In this light, then, the role of NbS in ‘building back better’ becomes increasingly important.

The ‘build back better’ (BBB) concept, first coined in 2006, became so widespread in its use and adoption that it became the second half of Priority 4 of the Sendai Framework for Disaster Risk Reduction 2015–2030 (Fernandez and Ahmed, 2019). It speaks to opportunities for survivors of disasters to change or improve policies and practices that lead to increased vulnerability. The concept by definition requires action post disaster; however, this paper expands this to include simply a ‘build better’ lens, which is critical for ‘developing states’ such as Jamaica and their continued expansion into marine and coastal areas. This paper presents an overview of three of the top marine and coastal NbS being used in Jamaica: protected area management (Special Fishery Conservation Areas (SFCAs)); mangrove restoration; and coral restoration. It briefly reviews their current application in Jamaica before arguing that these conservation projects that traditionally focused on biodiversity have a broader purpose in ‘building back’ as Nature‐based Solutions to disaster resilience.

## Methodology

### Case study: NbS inventory in Jamaica

An inventory of climate and biodiversity‐related projects in Jamaica was developed between September and November 2021 through an online survey and targeted (direct communication and requests for information) stakeholder interviews—part of an initiative of Global Affairs Canada. The online survey was implemented as an eight‐question Google Form that was disseminated widely across various networks. A total of 43 projects relevant to the topic were sent in for inclusion in the inventory. The semi‐structured interviews were based on the Google Form questions. These questions sought to identify the key characteristics—location, funding amount, dates, partners, objectives, and achievements—of projects that were: (i) relevant to the themes of NbS, climate change, and/or biodiversity; (ii) conducted within the past 10 years (2011‐21); and (iii) more than CAD 20,000 in value. Projects were categorised using multiple criteria, including project status (in the pipeline, ongoing, or ended), budget range, IUCN Category of NbS^2^ (eco‐DRR, ecosystem‐based adaptation, green infrastructure, holistic regenerative landscape management, natural infrastructure, and natural solutions), geographic scope of project activities (marine, terrestrial, or both), and the main focal area of activities (which fell into the categories of agroforestry, biodiversity enhancement, capacity‐building, sustainable finance, climate‐smart agriculture, coral restoration, wetland restoration/eco‐DRR, urban ecosystem‐based adaptation, and protected area management).

A total of 40 institutions, including government entities, non‐governmental organisations (NGOs), private sector bodies, academia, and bilateral and multilateral funding agencies, were contacted for information. This was supplemented by interviews with 17 key project implementers and stakeholders in the climate change and biodiversity sectors. These approximately one‐hour‐long, virtual interviews were open, seeking to collect more information on practical experiences of implementing NbS in Jamaica. Data from the online form and interviews were collated in a Microsoft Excel inventory database and assessed qualitatively using interview notes. Information was collected for 99 projects in total.

### Case study: Jamaica's SFCA network

This case study uses a mixture of a literature review and primary research to outline the current state of the SFCA network in Jamaica. A SFCA network review was conducted between August 2019 and February 2020, involving a semi‐structured interview approach, with the managers of all 18 SFCAs in the country. Interviews were performed by the primary author virtually or in person where possible and took approximately one hour to complete. A total of 12 managers were interviewed (as some are responsible for more than one sanctuary). A total of 42 questions were developed based on the guiding criteria for establishing SFCAs in Jamaica (Fisheries Advisory Board, 2002), as well as on literature on best practices and lessons learned from marine protected areas (MPAs) globally (Green, White, and Kilarski, 2013; Edgar et al., 2014; Weeks et al., 2014). The survey captured information on physical characteristics, management and operations, ecological status, human impacts, and social factors. The results were summarised and analysed using Excel spreadsheets, creating descriptive statistics.

### Case study: mangroves and mangrove restoration in Jamaica

The University of the West Indies (UWI) has planned and executed or assisted with several pilot mangrove restoration sites between 2015 and 2020. The selected sites all exhibited the potential for rehabilitation, having a non‐degraded forest nearby, indicating the localised effects of the disturbance.

Falmouth, located at the Winns Morass conservation site and managed by the National Environment and Planning Agency (NEPA), is a former informal housing parcel with marl/limestone and concrete rubble material that was deposited in the 1980s, spawning a two‐metre‐high and dry environment. This fill material was excavated from the site by surface scraping (using a 20‐ton excavator) to create a 0.5–2.0 per cent slope on the forest floor, which was then agitated subsequently by the excavator teeth to reduce the heavy equipment compaction effects. The material was carted off to an approved dump site in December 2020.

Restorative work at the Portland Bight Protected Area (PBPA), implemented by the NEPA, involved the creation of a two‐metre‐wide canal parallel to an unplanned roadway to facilitate fishing operations in the Portland Cottage community. This roadway was blocking tidal flow into the mangrove forest. In addition to the canal, a one‐metre‐wide culvert though the roadway was installed. This renewed the entry of seawater beneath the previously impenetrable roadway, which travelled into the canal, facilitating the required hydrological liberation needed to sustain mangrove seedlings in the area.

Additional sites were located at Bogue, Lilliput, and Palisadoes. Bogue and Lilliput were previously reclaimed by informal and unplanned works. While no channels or culverts were created, slope alterations were used to allow optimal tidal wetting and to restore their historic tidal hydrology. The Bogue and Lilliput sites had material removed to match the slope of an adjacent forest (Trench, 2021). At the Palisadoes site, substrate was reintroduced (following its removal during road construction) to create an appropriate base for mangrove seedlings to be planted. The recreated or reshaped mangrove areas were sloped between 0.5–5.0 per cent in these cases. This slope gradient range was derived from topographic surveys in several undisturbed mangrove forests in Jamaica (Trench, 2021).

The pilot sites were assessed for changes in vegetative and some edaphic and physico‐chemical features between the time of the restorative inputs and 24 months later. Several parameters—tree height, canopy cover, tree density, soil organic matter, water depth, salinity, slope, and tidal height—were used to appraise the different mangrove sites for changes in key ‘restoration benchmarks’. Monitoring of the sites occurred primarily in permanent 10 × 10 ‐metre monitoring plots, noting changes in the various indices over the monitoring period. Statistical tests were also conducted on the data, to compare month zero (restoration inputs period) to 24 months into the rehabilitation period.

## Results and discussion

### Review of NbS for disaster resilience in Jamaica

**Figure 1:**
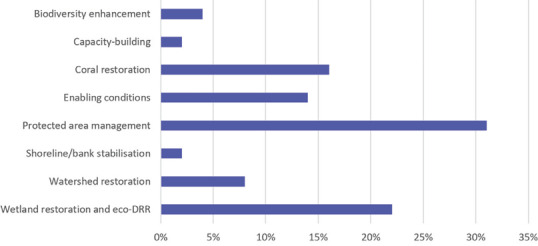
Main thematic areas of marine and coastal NbS identified in Jamaica between 2011 and 2021 **Source**: Lee and Hall (2021).

There is very little in the academic literature on the use of NbS in Jamaica. A rapid review of publications that look at NbS, ecosystem‐based adaptation, or eco‐DRR in Jamaica produced few relevant works; the majority focused on reports from projects or examined the theme from a governance perspective. The results of the rapid review are not necessarily reflective of a dearth of NbS being applied in Jamaica; as demonstrated by the national inventory (Lee and Hall, 2021), several projects could be considered as NbS, whether they used the term or not. One should note that of the 99 projects identified, there were a few large projects, including establishing trust funds, that supported multiple small‐scale NbS activities across different sites. Of these 99 projects, 47 were implemented in the marine/coastal zone or were both terrestrial and marine/coastal. Of these interventions, 31 per cent were categorised under the main theme of ‘protected area management’, 22 per cent under ‘wetland restoration’, and 16 per cent under ‘coral restoration’ (see Figure 1).

While few marine/coastal projects mentioned a DRR objective, it can be assumed that co‐benefits for resilience could be realised once interventions are successful in the long term. None of the projects identified had long‐term monitoring programmes in place that collected data to prove concretely the existence of resilience co‐benefits. It was also found that many of the coral restoration projects occurred in protected areas, implemented as a strategic action to improve biodiversity. A few mangrove restoration projects also took place within protected areas. The role of protected areas, mangroves, and coral reefs in supporting coastal resilience is well documented in the literature (IUCN, 2020).

The remaining subsections contain case studies in two of these categories—protected area management and wetland restoration—and provide an overview of coral restoration work in Jamaica.

### Case study: Jamaica's SFCA network

**Figure 2:**
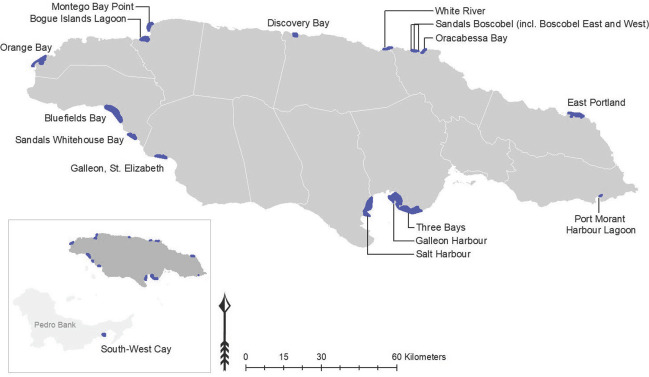
Location of the existing 18 SFCAs in Jamaica **Source**: authors, with input from The Nature Conservancy.

In response to the dramatic decline and near collapse of the Jamaican fishing industry from the 1950s, key stakeholders in the industry made early propositions for marine reserves in the 1970s. In 2008, they formed the Fisheries Advisory Board, which assumed responsibility for, among other things, establishing a network of fish sanctuaries around the island (Munro, 1983; Aiken and Haughton, 1987; Aiken et al., 2011). As of 2021, there are 18 declared SFCAs around Jamaica (see Figure 2).

Some studies do exist on Jamaican MPAs, looking at the use of artificial reefs, financing, ecosystem services, well‐being bundles, and the potential for sustainable tourism (Roach et al., 2004; Reid‐Grant and Bhat, 2009; Clegg, 2015; Chan, 2017). But to the best of our knowledge, no peer‐reviewed articles exist that speak to the success or lack thereof of Jamaica's SFCAs and their ability to meet management objectives. Data on fish biometrics and habitat status are being collected in some SFCAs, mostly in an ad hoc manner, based on funding availability (such as from projects); usually it remains within the managing bodies themselves or as grey literature within other government agencies or organisations. This information was not available for this review, nor does this case study seek to establish whether or not SFCAs have been successful. Instead, this case study examines different factors for success as identified in the literature, to provide some insight into the potential success of the network of SFCAs. It postulates that the success of SFCAs should be measured in terms of more than just their provisioning ecosystem services (as measured through fish biometrics); it needs to be gauged, too, in terms of their larger role in both ecological and social resilience in the context of ‘building back’.

Edgar et al. (2014) identify five characteristics—no‐take, enforced, old, large, and isolated (NEOLI)—that contribute to an effective MPA. Table 1 assesses them as they pertain to Jamaican SFCAs, revealing substantial variations. In this review, the number of years since active management was examined rather than age since designation. Edgar et al. (2014) indicate that an effective MPA should include four of the five characteristics. Based on the review, Jamaican SFCAs already face challenges in terms of the ‘large’ and ‘isolated’ NEOLI characteristics and vary with respect to others.

**Table 1:** A comparison of how Jamaican SFCAs are represented in each of the characteristics identified by Edgar et al. (2014)

NEOLI characteristic	Result from the SFCA review
No‐take	All are legally zoned as no‐fishing zones with the purpose of replenishing fish stocks.
Enforced	Level of enforcement was not directly measured but was identified as a challenge by 9 of the 18 SFCAs.
Old (more than 10 years)	Range from 4 years (White River SFCA) to 29 years (Bogue Lagoon and Montego Bay Point), at the time of the interview.
Large (more than 100 square kilometres (km^2^))	Range from 0.8 km^2^ (Oracabessa Bay) to 13.4 km^2^ (Bluefields Bay).
Isolated	All SFCAs border the coastline (or an island in the case of Bird Cay), with special note made of two clusters (three or more SFCAs in close, less than seven kilometres, proximity to each other): the Oracabessa Bay, White River, and Boscobel SFCAs; and the Salt Harbour, Galleon Harbour, and Three Bays SFCAs.

**Source**: authors.

The NEOLI characteristics identified by Edgar et al. (2014) are not the only conditions for success that have been outlined by other authors. Social or community involvement in establishing and managing SFCAs is also a factor cited for success (Weeks et al., 2014; Giakoumi et al., 2018). All Jamaican SFCAs specify that fisher‐folk were consulted before the establishment of a SFCA, although the level of consultation varied across sites. At a minimum, and in only one instance, the consultation was informal and described as ‘some discussion’, whereas others, mostly concerning the newer SFCAs, entailed extensive or systematic involvement of community members, even beyond fishers. In considering the design of the SFCA and the location of boundaries, local input was the most common determining factor (15 SFCAs) followed by ecological factors (nine SFCAs). Of the 18 SFCAs, management (sole or in partnership) is in the hands of a community‐based organisation in four cases, a local NGO or not‐for‐profit organisation in 13, and the government in one. The involvement of communities from the onset could be one of many possible contributing factors to the reported increase in compliance with the SFCA since designation, with most (11 SFCAs) reporting that the ‘majority of local fishers currently support the SFCA’ rules.

This review highlighted that Jamaican SFCAs meet design or social criteria for success to different extents and tend naturally to apply a fisheries lens when considering metrics of success. However, SFCAs can provide numerous co‐benefits that are not typically accounted for in monitoring. In the Bluefields Bay SFCA, both provisioning (fisheries) and cultural (heritage) ecosystem services were identified by community members as important services associated with the SFCA (Chan, 2017). It is within this wider socioecological and ecosystem services realm that the role of SFCAs in disaster resilience becomes clear.

Functioning marine managed areas (MMAs) can provide multiple co‐benefits that support ‘building better’ in the context of disaster resilience. With respect to climate resilience and climate change mitigation, which is undeniably linked to disasters, MMAs (such as SFCAs), while not initially designed with climate change resilience values in mind, have proven to be effective measures in helping to meet these goals (Mcleod, Salm, and Green, 2009). The ocean acts as a climate regulator, absorbing carbon and additional heat generated by human activities, while also releasing oxygen (Roberts et al., 2017). A strong culture, social cohesion, and good economic standing are important contributors to post‐disaster social resilience (Enzi et al., 2017; Mabon, 2019). Post establishment, 13 SFCAs reported having facilitated some form of social intervention activity revolving around alternative livelihoods (11 SFCAs), SFCA‐related activities such as employment as wardens or data collection (nine SFCAs), or life and livelihood support such as improved living conditions or equipment (one SFCA).

On the ecological side, conserving the key ecosystems that help to maintain biodiversity, enabling marine systems to act as heat absorbers and carbon pumps, helps to strengthen the general climate resilience of an area (Lecerf et al., 2021). Coral restoration efforts have been or continue to be conducted in several of the SFCAs, involving, inter alia, artificial reefs and a variety of nursery types and forms of research—discussed further in the ‘coral restoration’ section of this paper. SFCAs adopt, deliberately or otherwise, a landscape approach to conserving key ecosystems that help to maintain biodiversity, thus allowing for multiple ecosystem values that permit the realisation of mitigation.

The results of this review, combined with insights from interviews performed for the national NbS inventory, paint a much more impactful picture of SFCAs beyond simply increasing fish stocks. They have the necessary characteristics and have laid the foundation for facilitating activities that can increase social and ecological resilience. They are often the sites for research on scalable NbS for disaster resilience, such as different types of mangroves and coral reef restoration techniques, and coastal vegetation enhancement experiments. They also serve as platforms to help educate the public in the factors relating to NbS, disaster resilience, and the environment in general. If stakeholder engagement is a prime objective for the protected area, then it also helps communities to have a sense of ownership in their roles as environmental stewards. All of these factors support the argument to ‘build back’ with community‐based protected areas with the long‐term aim of improving the resilience of an area.

### Case study: mangrove restoration activities in Jamaica

Jamaica had an estimated 11,674 hectares of coastal wetland area in 2010, which mainly comprised mangrove forests; the majority (82 per cent) were found in the southern parishes and were typified by a dominance of black mangrove (*Avicennia germinans*) (National Environment and Planning Agency, 2013b; Torres Ortega et al., 2019). Based on a review of satellite images, Jamaica lost more than 2,000 hectares of mangroves between 2005 and 2010. This loss has been attributed to numerous factors, such as coastal development, especially tourism and housing, formal and informal (particularly in Falmouth and Portmore), improper sewage treatment, and solid waste associated with settlements (National Environment and Planning Agency, 2010; Trench, 2021). Agricultural and infrastructural works have also contributed to mangrove degradation, as seen with the pollution impacting the Port Royal mangroves and along the Palisadoes tombolo in Kingston (Alleng, 1998). Climate change is also expected to affect mangrove forests negatively through, among other things, increased damage owing to storm events (National Environment and Planning Agency, 2010; Torres Ortega et al., 2019).

Mangrove restoration is a return of all pre‐existing conditions, before a negative and irreversible disturbance, whereas rehabilitation may be considered as a route towards restoration, where the objective is to improve existing conditions, to what can be considered as ideal/original or near ideal (Lewis, III, 1990, 2005). Jamaica's mangrove restoration history is relatively short, and not well documented, residing in technical reports and theses primarily; mangrove restoration ‘attempts’ employing mangrove gardening techniques were more common. The limited number of hydrology‐ and ecology‐based restoration attempts may be regarded as successful or ‘en route’ to restoration (Trench, 2019).

Restoration and rehabilitation activities were piloted by one of the authors at different sites across Jamaica. One was the rehabilitation of 2.0 hectares of a degraded mangrove forest located in the PBPA along Jamaica's southern coast, which had suffered damage during Hurricane Ivan in September 2004, coupled with the unplanned construction of a temporary road, resulting in restricted hydrological connections within the forest. Restorative activities within the PBPA by the NEPA in 2012 improved the hydrological connections and facilitated tidal flushing and natural seedling regeneration over time (National Environment and Planning Agency, 2013a). With additional adaptive management inputs to keep out feral goats, the assessments conducted two years later revealed 40 per cent survival of planted seedlings, but a 350 per cent rise in volunteer seedling recruitment and impressive mangrove seedling growths, as well as the development of prop and breathing roots, increasing in height by more than 300 per cent over the period (National Environment and Planning Agency, 2013a).

Another identical but younger project (less than two years old) in Falmouth Trelawny, at the Winns Morass conservation site, is managed by the NEPA. Twelve months after restoration activity to remove disturbed sections, mangrove seedlings were naturally recruiting and more than 0.5 metres tall, with tidally active estuarine waters around their roots.^3^ Although the data would not be similar enough to reference, the results were promising and indicated that these sites were en route to restoration. Restoration pilot studies by the UWI and/or the NEPA two years after planting reported expected results for ‘restoration’ sites, with increases in key indices, including tree height, number of nodes and diameter at breast height, flowering and fruiting plants between 18 and 24 months, seedling recruitment, salinities ranging from 20–35 parts per thousand, higher mean water level, and an increase in soil organic content (Trench, 2021).

In the case of the rehabilitation activities mentioned above, it is significant to note the role of a key road area of Falmouth in DRR, owing to the absence of mangroves in adjacent areas. The area changed from a calm mangrove‐dominated shoreline to a location with higher wave energy, following the construction of a major shipping port immediately north of the roadway. The port's construction entailed the relocation of more than 147,947 coral reef colonies (Jamaica Environment Trust, 2013) in water depths of 2–20 metres, dredging the area to be a suitable depth for the mooring and turning of cruise ships, and the loss of many acres of seagrass and more than 40 hectares of mangrove, without any clear and documented mitigation (Jamaica Environment Trust, 2013). The National Works Agency (2016) spent more than JMD 15 million on installing rock revetments along the roadway in 2016, to address sections eroding and posing serious risks to life and property along the then busy thoroughfare. Revetments were needed specifically in the sections that lacked mangrove shorelines, as compared to the mangrove‐lined edges that showed minimal coastal disturbance.

Of the more than 200 functions of mangroves worldwide, it is well accepted that their roles in storm surge and wave attenuation are some of the most valuable, as was brought into clearer focus following the tsunami in Southeast Asia on 26 December 2004 (Dahdouh‐Guebas et al., 2005). The World Bank's *Forces of Nature* study (Torres Ortega et al., 2019) highlighted that Jamaica's mangroves play an important part in shoreline protection, providing more than USD 32.6 million in annual flood reduction benefits to built capital (on average around USD 2,500 per hectare per year). In addition, without mangroves, the total number of people flooded every year could increase by 10 per cent. In terms of site‐level assessments, mangroves were found to reduce wave heights by between 13 and 68 per cent, and wind speed by between 11 and 80 per cent. Protection services and value varied depending on the location within Jamaica, increasing in value in areas with a higher population or more frequent and larger storm surges. For example, mangroves at Bogue Lagoon (Montego Bay) offer high shoreline protection ecosystem services given the proximity of critical road infrastructure and dependence on mainstream and alternative tourism. Although Portland Cottage lacks these factors, the population in the area is most at risk and vulnerable; the mangroves present at this site thus offer strong protection services with respect to life and livelihoods and reduce the costs to government in the event of a serious disaster. The majority (67–75 per cent) of persons in all study sites expressed willingness to become involved in mangrove restoration activities.

The restoration of mangrove ecosystems is beneficial if one wants to take advantage of the DRR services that mangroves offer. As highlighted in the example of Falmouth, ‘building back’ can also apply to developmental ‘disasters’ that remove the ecosystem services for surrounding lives and livelihoods. The successes of these pilot projects, coupled with methods to identify the restoration potential of sites based on environmental factors, pave the way to a promising future for Jamaica's mangrove conservation and restoration, and will ensure coastal protection safeguards for the numerous livelihoods that exist behind and adjacent to these forests (Trench, 2018; Torres Ortega et al., 2019). These projects are poised to scale up the enhancement and rehabilitation of mangroves as NbS island‐wide, as part of improved forest management under the Forestry Department.

### An overview of coral restoration activities in Jamaica

Coral reefs provide infrastructural benefits for DRR, being the first line of defence against erosion and flooding, and helping to deflect wave energy during storm events (Reguero et al., 2018). Fringing reef crests function similar to low crested breakwaters, protecting the shoreline, and are the main source of sand, helping to build up beaches (Bellwood, 1995). Reefs also generate carbon sequestration benefits and are regulatory agents for organic carbon, making indirect contributions to improved coastal resilience (Roger and Brent, 2012).

There is strong evidence that the Caribbean's coral reefs are in jeopardy (Jackson et al., 2014; Souter et al., 2020), highlighting the need for interventions that will improve conditions that help to increase live coral cover and structural complexity for at‐risk reefs. As such, reef restoration has been proposed as an NbS for many tropical marine environments, supplementing natural coral populations and in the long term, theoretically, enhancing ecological services. Given that reefs are slow‐growing and face so many threats, the response times for significant changes in broadscale habitat complexity and improved biodiversity in an area will not be within the scope of any typical restoration project. There is limited evidence of the long‐term ecological success of reef restoration activities (Hein et al., 2020). However, it is theorised that successful reef restoration activities in combination with other broader reef resilience management strategies, such as threat reduction and marine spatial planning, will result in augmented coastal resilience in the long run if the enabling conditions are ideal for more resilient reefs to continue to grow (Hein et al., 2020).

The increase in the implementation of reef restoration projects across Jamaica within the past 10 years has not necessarily been directly to boost resilience; activity has been driven more by a sense of urgency to improve benthic habitat biodiversity following catastrophic losses of scleractinian coral after various disasters. The NbS inventory and assessment revealed eight notable coral restoration projects since 2011, with the majority (75 per cent) being small one‐off projects (USD 25,000–50,000) with no long‐term monitoring system in place (Lee and Hall, 2021). These do not include smaller (less than USD 20,000) experimental or ad hoc coral restoration activities being conducted by organisations or individuals. The study also highlighted that most coral restoration projects occurred within protected areas that had a clear, operational management body (see Box 1) (Lee and Hall, 2021). Occasionally, some restoration work has also been done to offset the negative effects of dredging and other coastal infrastructure development projects, such as the construction of Falmouth Pier (Smith Warner International, 2018) and the widening of the ship channel for Kingston Harbour (Trench et al., 2014). Box 1 (above) details the results of a sample of reef restoration efforts that have been implemented in MMAs across the island.

**Box 1. General observations of more notable coral restoration efforts in priority biodiversity areas of Jamaica**

**Discovery Bay SFCA:**

- Implemented and managed by the UWI's Center for Marine Sciences (UWI‐CMS) and the Alloa Fisherman's Co‐Operative.
- Pioneer in the Caribbean in reef restoration, with in‐situ and ex‐situ nurseries established and multiple grant‐based projects and academic studies implemented due to the UWI's Discovery Bay Marine Laboratory being situated within the boundaries of the MMA.
- Facilitates partnerships with strategic entities, such as academia and relevant government agencies.
- Implemented artificial reef structures to supplement natural reef habitats.
- Has led research to identify resilient coral phenotypes and understand the resilience of local coral populations, suitable and cost‐effective coral culture techniques, sexually reproduced recruits, and developed a coral rearing programme for spawners.
- Does work to understand the primary threats to the overall health of reefs, notably, water quality monitoring programmes and the identification of point sources of pollution.
- Level of success of in‐water restoration efforts is variable, depending on prevailing environmental conditions such as water quality, including sea surface temperatures, the capacity of project staff, and availability of funds.
- Important findings/recommendations from restoration efforts include outplanting Acropora spp. coral at Back Reef habitats, as these fast‐growing, branching corals work well with Back Reef zones as wave breakers.

**East Portland SFCA:**

- Restoration projects are managed by the Alligator Head Foundation, an emergent and now leading force for reef restoration in Jamaica and the wider Caribbean.
- Facilitates partnerships with strategic entities.
- An ongoing research site for the UWI‐CMS.
- Has worked with an in‐situ and ex‐situ nursery, focusing mostly on in‐situ nursery activities.
- Has project proposals in the pipeline for capacity‐building with regard to ex‐situ facilitated asexual and sexual reproduction of resilient species.
- Has led significant efforts to raise public awareness and engage surrounding communities in reef restoration efforts.
- Recent studies by The Nature Conservancy (TNC) show that this should be a priority site for restoration work due to the likelihood of environmental conditions enabling growth in the future (Chollett et al., 2022).
- Has experienced varying levels of success in relation to the survival and growth rates of coral fragments. There is a long‐term monitoring programme in place, but the frequency of data collection is unknown and data are not frequently reported or published.

**Oracabessa SFCA:**

- Restoration projects are managed by the Goldeneye Foundation, the Oracabessa Marine Trust, and the Saint Mary Fisherman's Cooperative.
- Engaged in ongoing work with in‐situ coral nurseries that are maintained by fishers from surrounding communities who are also employed by the SFCA management body as wardens and coral gardeners.
- An ongoing research site for the UWI‐CMS.
- Has been successful in facilitating and maintaining community engagement and stakeholder buy‐in.
- Restoration efforts have been highlighted by local and international media outlets repeatedly.
- Recent studies by the TNC show that this should be a priority site for restoration work due to the likelihood of environmental conditions enabling growth in the future (Chollett et al., 2022).
- Fairly successful sanctuary management and restoration efforts, given anecdotal evidence of increased fish bio‐mass, abundance, and biodiversity (richness) within the sanctuary.

**White River SFCA**:

- One of the younger SFCAs, but it has restoration projects that are managed by the White River Marine Association.
- Engaged in ongoing work with in‐situ coral nurseries that are maintained by fishers from surrounding communities who are also employed by the SFCA management body as wardens and coral gardeners.
- An ongoing research site for the UWI‐CMS.
- Fairly successful restoration efforts, as evidenced by project reports (growth rate and number of live fragments outplanted). The survival rates of fragments outplanted in the wild are unknown.

**Portland Bight Protected Area**:

- One of the few sites on the south coast that has seen coral restoration efforts implemented in a few areas.
- Restoration efforts are managed by the Caribbean Coastal Area Management Foundation.
- An ongoing research site for the UWI‐CMS.
- Had in‐situ coral nurseries that were maintained by fishers from surrounding communities who were also employed by the SFCA management body as wardens.
- Success of restoration efforts has been variable due to sedimentation and other water quality issues (anecdotal evidence).

**Montego Bay Marine Park**:

- First‐ever MPA declared in Jamaica, located adjacent to a tourism hub (with tourist traffic) and the large city of Montego Bay.
- Restoration projects managed by the Montego Bay Marine Park Trust.
- Success of restoration efforts has been variable due to sedimentation and other water quality issues (anecdotal). Efforts are also not ongoing, as a funding and resource gap needs to be filled to ensure consistency of coral nursery operations and maintenance.

**Bluefields Bay SFCA**:

- Restoration projects are managed by the Bluefields Bay Fishermen's Friendly Society and NGOs.
- Restoration efforts have not been maintained consistently, despite capacity being developed to do so, owing to ongoing tensions between fishers and the SFCA management body, water quality issues, and insufficient funding to retain trained wardens and coral gardeners (except during the lifetime of projects).
- Success of restoration efforts is variable. Survival rates of outplanted species are unknown as no follow up has been done post projects.

**Port Royal Cays**:

- Restoration efforts have happened in the Port Royal Protected Area (Rackham's Cay).
- Major restoration and relocation efforts implemented to mitigate the loss of benthic species after dredging to expand the shipping channel were initially successful (Gayle, Wilson‐Kelly, and Green, 2005), but slow growth and a high rate of mortality at deeper depths experienced in the long term (Trench et al., 2014).

**Source**: authors' literature review and interviews with key stakeholders (Lee and Hall, 2021).

Several uncertainties or threats, such as pollutants, temperatures, and diseases, are associated with coral restoration and beyond implementers' control, which prevent them from being a ready‐made solution for climate and disaster resilience. In Jamaica, coral restoration activities will only be successful if the causes of reef degradation are reduced or removed (National Environment and Planning Agency, 2021). Consequently, threat reduction is an important management activity to employ to complement the implementation of NbS. Restoration efforts can help to kickstart system recovery in areas where there are natural barriers to coral recruitment. The recommended approach is to use coral restoration as a complementary effort to local management strategies to aid recovery following disturbances (indirectly contributing to improved resilience) (Hein et al., 2020).

## Conclusions and recommendations

Although many of the NbS activities in Jamaica are not driven primarily by disaster resilience and BBB, but more by biodiversity concerns, the co‐benefits are clear not only in the global literature, but also in anecdotal, local‐level studies. Jamaica has made substantial progress in tapping into the eco‐DRR benefits of marine and coastal protected area management, mangrove restoration and rehabilitation, and coral restoration, and exhibits good potential for work in the future.

Natural systems are usually well adapted to disturbance regimes, such as storms, episodes of drought, and flooding, and have yielded DRR and other benefits that ‘grey’^4^ or hard infrastructural engineering products try to achieve (Francis, 2013). There is an increasing amount of evidence that coastal management strategies that align coastal engineering and ecological objectives deliver a multiplicity of benefits, including disaster resilience (Bridges et al., 2014; Kurth et al., 2020; González‐Dueñas and Padgett, 2021). These engineering solutions are often designed to address current ecosystem conditions and consider exposure and multi‐hazard risks. However, an approach that relies heavily on conventional grey infrastructure may not be realistic for Small Island Developing States for a number of reasons. These include the fact that achieving the desired level of coastal protection may be iterative and costly to install and maintain (Temmerman et al., 2013). There is also the risk of negatively affecting coastal ecosystems (Gittman et al., 2016), rendering these solutions unsustainable. Temmerman et al. (2013) postulate that ecosystem‐based approaches to coastal protection may be more ideal and cost‐effective. Research also shows that stakeholders prefer to support NbS as compared to grey infrastructure (Deeley, 2020). In cases where climate change risks are more acute, a combination of grey and green/blue infrastructure could also be examined to address short‐ and long‐term challenges (Kurth et al., 2020). The continued exploration and implementation of complementary NbS actions are highly recommended as the most practical approaches to coastal management in Jamaica.

The findings of this paper indicate that although much work is being done in Jamaica with respect to NbS, climate change, and resilience, documentation of activities in the public domain is lacking. The same is true for work being done through SFCAs and mangrove and coral restoration. While the success of the well‐established Jamaican SFCA network is yet to be determined, this case study highlights that some contributing success factors are present within it. The paper emphasises the important role that SFCAs have to play in social and ecological resilience. Despite a relatively short mangrove restoration history, the island has a number of pilot projects that have produced promising results. There has also been a positive, general transition from the notion of ‘mangrove gardening’ to rehabilitating mangrove forests for coastal protection benefits. Keystone projects such as the one implemented by the World Bank (Torres Ortega et al., 2019) (not published academically) clearly demonstrate the part that mangroves play in disaster resilience, making the case for more consideration of mangrove removal in future developments. Similarly, there are numerous coral restoration activities and projects being implemented in Jamaica with varying success. With leading research organisations on the island, there is great opportunity to collect scientific and robust data to guide restoration activities in the future.

In the context of ‘building back’, more work should be done to make a stronger case for NbS and their disaster resilience benefits. Project implementers and donors should encourage the publishing of results to increase awareness of how NbS can contribute to climate and disaster resilience, and create a bountiful database of citable, peer‐reviewed, and reusable information to guide decision‐makers and key players in BBB. The areas of focus for research could include gathering and publishing scientific evidence of NbS successes (with respect to SFCAs and mangrove and coral restoration) in Jamaica, identifying and valuing resilience co‐benefits, better approaches for incorporating climate change adaptation in management and restoration action plans (O'Regan et al., 2021), and identifying improvements to designs and methodologies based on literature and primary research (Green, White, and Kilarski, 2013; Edgar et al., 2014).

The demands that resilience as an objective places on natural resource management efforts are numerous; a multi‐pronged approach is required to ensure that solutions can fulfil all needs necessary. Therefore, NbS have to be well thought out before implementation to maximise improvements in natural resilience to climate change effects. Solutions need to be designed that are issue‐specific, occur at an appropriate spatial scale, encompass sustainable actions geared towards long‐term management, and are not in major conflict with ecological values (IUCN, 2020). For research, policy, and scaled NbS to support real change, interventions must be well planned, possess long‐term monitoring features, and be targeted as place‐based actions of people in partnership with nature. In general, a better understanding of the coupled feedback loops between human and ecological coastal systems, including an understanding of the stress thresholds of ecosystems, would also be informative for maximising the benefits to both, and help to inform adaptive management measures for NbS.

The findings of this review reveal that, a lack of published literature notwithstanding, there is a plethora of practical experience to be garnered and collated to streamline better biodiversity and NbS in BBB. This study can be used to help steer critical strategic actions for these solutions to be integrated into more climate and biodiversity initiatives across Jamaica. Improving ecosystem conditions by incorporating NbS actions in policies, development plans, and governance documents, and mainstreaming the implementation of the central tenets of NbS, would augur well for the achievement of Jamaica's sustainable development plans under the fourth national goal of Vision 2030: ‘Jamaica has a healthy natural environment’.^5^

## Acknowledgements

The authors would like to acknowledge the important contributions to this paper made by the Government of Jamaica, the World Bank Group, and all the researchers and authors associated with the PROFOR (Program on Forests) project entitled ‘Assessment and Economic Valuation of Coastal Protection Services Provided by Mangroves in Jamaica’. Thanks are also extended to Global Affairs Canada for the use of the data collected under the project ‘Assessment of Nature‐based Climate Resilient Projects in Jamaica’. And finally, thanks to the staff members of the University of the West Indies who provided invaluable support in developing the paper.

## Data availability statement

The data that support the findings of this study are available from the corresponding author upon reasonable request.

## Endnotes

1 See https://www.iucn.org/theme/nature-based-solutions (last accessed on 28 April 2022).

2 The IUCN's definition of NbS was used to assess whether or not projects could be considered for the inventory.

3 Authors' observation, Camilo Trench, 2021.

4 ‘Grey’ infrastructure refers here to the human‐engineered infrastructure for coastal, freshwater, and other resources, such as breakwaters, dykes, groynes, reservoirs, and wastewater treatment plants.

5 For more information, see https://jis.gov.jm/information/get-the-facts/vision-2030-jamaicanational-goal-4/#:~:text=Therefore%2C%20the%20fourth%20National%20Goal,and%20adaptation%20to%20climate%20change (last accessed on 29 April 2022).
